# The hidden cost of stress: knowledge hiding, innovative behavior, and the buffering role of leader–member exchange

**DOI:** 10.1186/s40359-026-05095-z

**Published:** 2026-07-02

**Authors:** Ji Hoon Lee, Jeeyoon Jeong, Jung-Mi Park, Steven J. Karau

**Affiliations:** 1 Wonkwang University College of Business, 460 Iksandae-ro, Iksan Jeonbuk, 54538 South Korea; 2 Nagoya University of Commerce and Business NUCB Business School of Management, Sagamine-4-4 Komenokicho, Nisshin, Aichi 470-0193 Japan; 3 Southern Illinois University Carbondale College of Business and Analytics, 1025 Lincoln Drive, Carbondale, IL 62901 USA

**Keywords:** Strain-related job stress, Knowledge hiding, Innovative behavior, Leader–member exchange, Conservation of resources theory

## Abstract

Grounded in conservation of resources theory, this study examines how strain-related job stress is associated with employees’ innovative behavior through knowledge hiding and whether leader–member exchange quality weakens this indirect relationship. Drawing on two-wave time-lagged survey data from 496 employees in South Korea, we found that strain-related job stress was negatively associated with innovative behavior and positively associated with knowledge hiding. Knowledge hiding, in turn, was negatively associated with innovative behavior, partially mediating the relationship between strain-related job stress and innovative behavior. In addition, leader–member exchange quality moderated the relationship between strain-related job stress and knowledge hiding, such that the positive association was stronger when leader–member exchange quality was low rather than high. The conditional indirect relationship between strain-related job stress and innovative behavior through knowledge hiding was significant only under low leader–member exchange quality. These findings suggest that strain-related job stress may be associated with lower employee innovative behavior not only through internal resource depletion, but also through defensive regulation of knowledge access. The study contributes to research on job stress, knowledge hiding, and innovative behavior by identifying knowledge hiding as a social-behavioral pathway linking strain-related job stress to reduced innovative behavior and by highlighting high-quality leader–member exchange as a relational resource that may interrupt this pathway.

## Introduction

In contemporary organizations, employees’ innovative behavior is increasingly important for sustaining adaptability, problem solving, and competitive advantage. Innovative behavior refers to employees’ intentional generation, promotion, and implementation of new ideas within a work role, group, or organization [25, 41]. Prior research has emphasized that innovative behavior is not merely an individual cognitive activity, but also a socially embedded process that depends on information exchange, feedback, collaboration, and the recombination of knowledge across organizational members [2, 3, 32]. Thus, the circulation of knowledge within organizations is central to employees’ ability to recognize problems, develop novel ideas, and implement creative solutions.

At the same time, knowledge does not always flow freely within organizations. Although knowledge sharing can facilitate learning, creativity, and innovation, sharing knowledge may also expose employees to uncertainty, vulnerability, or loss of personal advantage [10, 27]. Employees may therefore intentionally withhold or conceal knowledge requested by others, a behavior known as knowledge hiding [9]. Knowledge hiding has been shown to undermine collaborative relationships, reduce trust, disrupt knowledge exchange, and impair important work outcomes [7, 8, 26, 42]. However, despite increasing research attention, knowledge hiding remains theoretically important because its antecedents and consequences are not yet fully understood, particularly in stressful work contexts where employees may become more protective of valued resources [23, 30, 38].

In this study, we focus specifically on strain-related job stress, reflected in employees’ psychological and physical tension associated with work, rather than on motivating challenge demands that may be appraised as opportunities for growth. Drawing on conservation of resources theory (COR; [20, 21]), we propose that strain-related job stress may be associated with knowledge hiding because stress places employees in a resource-protection mode. COR theory suggests that individuals strive to obtain, retain, protect, and build valued resources, and that stress occurs when these resources are threatened, lost, or insufficiently replenished after investment [18, 19, 20]. In the workplace, knowledge can function as a valuable resource because it allows employees to solve problems, maintain competence, influence work processes, and preserve status. However, sharing knowledge also requires time, attention, cognitive effort, and interpersonal exposure. Under strain-related job stress, employees may therefore perceive knowledge requests not simply as opportunities for cooperation, but as additional claims on already strained resources. From this perspective, knowledge hiding can be understood as a defensive resource-protection response through which employees attempt to conserve valued resources and avoid further depletion.

This perspective extends prior research on the relationship between job stress and innovative behavior. Existing studies have shown that job stress can undermine creativity and innovative behavior by consuming cognitive resources, increasing strain, and reducing employees’ capacity for exploration and experimentation [25, 29, 37]. However, stress research has also cautioned against treating all forms of stress as uniformly harmful. The challenge–hindrance stressor literature suggests that some demands may stimulate motivation and achievement, whereas others constrain growth and deplete resources [6, 28]. Accordingly, the present study does not examine challenge demands or motivating job demands. Instead, it examines strain-related job stress as a psychological and physical tension experience that is theoretically more closely aligned with resource depletion than motivational activation. We therefore argue that strain-related job stress may be associated with lower innovative behavior not only through internal strain-based mechanisms, such as emotional exhaustion, cognitive overload, or reduced intrinsic motivation, but also through a social-behavioral pathway involving defensive regulation of knowledge access.

Knowledge hiding is a theoretically distinctive mechanism in this process for two reasons. First, it captures an active knowledge-related response to stress rather than a general reduction in effort, withdrawal, or silence. When employees experience strain-related job stress, they may attempt to conserve their remaining resources by limiting the time, attention, and expertise they provide to others. Second, knowledge hiding may also restrict employees’ own innovative behavior. Innovative behavior depends on reciprocal information exchange, feedback seeking, collaborative problem solving, and the recombination of diverse knowledge inputs [2, 3, 41]. Employees who hide knowledge may weaken reciprocal exchange relationships, reduce coworkers’ willingness to provide help or information, and limit opportunities for idea refinement and implementation. Thus, knowledge hiding is not only harmful to others or to collective knowledge flows; it may also become a hidden barrier to the focal employee’s own innovative behavior.

We further examine leader–member exchange quality as a relational resource that may weaken the association between strain-related job stress and knowledge hiding. Leader–member exchange quality refers to the quality of the exchange relationship between supervisors and subordinates, reflecting the degree to which the relationship is characterized by mutual trust, respect, obligation, understanding, and work-related assistance [43]. Although leader–member exchange is broader than leader support narrowly defined, high-quality leader–member exchange can provide employees with supportive relational resources that are directly relevant to coping with stressful work demands. In high-quality exchange relationships, supervisors may provide employees with emotional reassurance, informational clarification, instrumental assistance, workload guidance, and interpersonal security. These resources may reduce the likelihood that stressed employees interpret knowledge requests as costly, threatening, or excessively burdensome.

When leader–member exchange quality is high, employees experiencing strain-related job stress may feel that they have sufficient relational resources, guidance, and supervisory assistance to respond to knowledge requests without fearing additional resource loss. High-quality leader–member exchange may also foster norms of openness and reciprocity, reducing employees’ perceived need to hide knowledge as a defensive coping response. In contrast, when leader–member exchange quality is low, employees may experience strain-related job stress as more isolating and threatening. Under such conditions, they may be more likely to conserve resources by withholding knowledge. Accordingly, leader–member exchange quality may interrupt the transition from resource threat to defensive knowledge regulation and thereby weaken the indirect association between strain-related job stress and innovative behavior through knowledge hiding.

The South Korean work context provides a meaningful setting in which to examine these relationships. Korean organizations are often characterized by relatively hierarchical relationships, strong expectations regarding supervisor guidance, and relationally embedded workplace interactions [12, 22]. In such contexts, employees’ responses to stress, decisions about whether to share or withhold knowledge, and perceptions of leader–member exchange quality may be shaped by supervisor–subordinate dynamics. Therefore, examining these relationships among South Korean employees allows us to consider how strain-related job stress, knowledge hiding, and leader–member exchange quality operate in a context where hierarchy and relational expectations may heighten the importance of supervisory relationships. At the same time, this context also requires caution in generalizing the findings to other cultural or institutional settings.

**Fig. 1:**
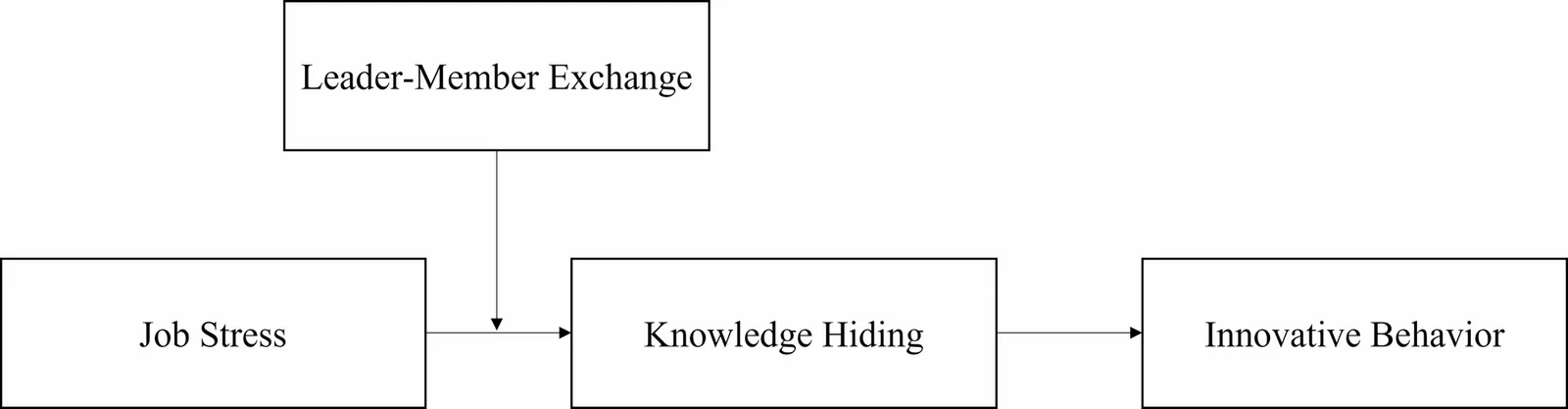
Hypothesized model

This study makes three primary contributions. First, it contributes to the job stress and innovative behavior literature by shifting attention from purely internal strain-based explanations to a social-behavioral pathway involving employees’ defensive regulation of knowledge access. Second, it contributes to the knowledge hiding literature by conceptualizing knowledge hiding not only as a counterproductive interpersonal behavior, but also as a potential resource-protection response under strain-related job stress. Third, it contributes to COR-based leadership research by identifying leader–member exchange quality as a relational resource that may interrupt the transition from resource threat to knowledge hiding. Figure 1 presents the proposed theoretical model.

### Theoretical framework

We draw on conservation of resources theory (COR; [20, 21]) to develop and organize our hypotheses. COR theory proposes that individuals strive to obtain, retain, protect, and build resources that they value. Resources include objects, personal characteristics, conditions, and energies that are valued in themselves or that help individuals obtain additional resources [20]. In organizational settings, these resources may include time, energy, attention, knowledge, autonomy, supportive relationships, high-quality leader–member relationships, and stable work conditions [18, 19]. Stress occurs when individuals perceive a threat of resource loss, experience actual resource loss, or fail to gain sufficient resources after investing resources.

COR theory is particularly useful for the present study because it explains how employees respond when they perceive that their available resources are threatened or depleted. Under strain-related job stress, employees may become motivated to conserve remaining resources and avoid additional resource expenditure [18, 20]. This resource-conservation logic has been used to explain a variety of workplace outcomes, including burnout, reduced performance, withdrawal, and knowledge-related behaviors [17, 19, 44]. However, to address the present research question, COR theory must be applied at the construct level. In our model, strain-related job stress represents a perceived resource threat; knowledge represents a valued work resource; knowledge sharing represents a potential resource investment; knowledge hiding represents a defensive resource-protection response; innovative behavior represents a resource-intensive and socially embedded work behavior; and leader–member exchange quality represents a relational resource that may buffer the stress–knowledge hiding link.

This construct-specific application of COR helps explain why strain-related job stress may be associated with knowledge hiding. Knowledge is valuable because it helps employees perform tasks, solve problems, maintain competence, and preserve influence within the organization. Yet sharing knowledge is not costless. Responding to others’ knowledge requests can require time, cognitive effort, emotional patience, and exposure of one’s expertise [9, 10]. When employees are already experiencing strain-related job stress, these requests may be appraised as additional demands on depleted resources. As a result, employees may hide knowledge to protect their remaining resources, avoid further workload, and retain control over valuable expertise. Thus, from a COR perspective, knowledge hiding can be understood as a specific resource-conservation strategy rather than simply a failure to share.

COR theory also clarifies why knowledge hiding may be linked to lower innovative behavior. Innovative behavior requires employees to invest substantial resources in idea generation, idea promotion, and idea implementation [25, 41]. It also depends on access to diverse knowledge, feedback, and collaborative support from others [2, 3]. Although knowledge hiding may help employees conserve resources in the short term, it may also reduce the social resources needed for innovation. Employees who hide knowledge may receive less reciprocal help, fewer informational inputs, and weaker collaborative support from coworkers. In this way, a behavior intended to protect resources may unintentionally restrict the resource flows needed for employees’ own innovative behavior.

Finally, COR theory provides a basis for the moderating role of leader–member exchange quality. Leader–member exchange quality is broader than leader support narrowly defined because it captures the overall quality of the supervisor–subordinate exchange relationship, including mutual trust, respect, obligation, understanding, and work-related assistance. From a COR perspective, high-quality leader–member exchange can function as a relational resource because it provides employees with access to supervisor understanding, guidance, influence, and assistance in dealing with work demands. These supportive relational resources may reduce employees’ need to rely on defensive responses such as knowledge hiding. Specifically, high-quality leader–member exchange may reduce threat appraisal by clarifying expectations, helping employees manage workload demands, and signaling that knowledge exchange is valued and safe. Thus, when leader–member exchange quality is high, the positive association between strain-related job stress and knowledge hiding should be weaker. Conversely, when leader–member exchange quality is low, employees may have fewer relational resources available and may be more likely to protect themselves by withholding knowledge.

Taken together, COR theory provides a focused framework for understanding the proposed model. Rather than using COR theory only to argue that stress is harmful, we use it to explain how strain-related job stress may redirect employees’ knowledge-related behavior toward defensive resource protection and how leader–member exchange quality may interrupt this process. This framework allows us to examine whether knowledge hiding functions as a social-behavioral pathway linking strain-related job stress to lower innovative behavior and whether this pathway depends on the quality of the leader–member exchange relationship.

### Hypotheses development

#### Strain-related job stress and innovative behavior

Job stress refers to employees’ psychological and physical strain resulting from work demands that are perceived as taxing or exceeding their available resources [24, 40]. In the present study, we focus specifically on strain-related job stress rather than challenge demands that may be experienced as motivating or growth-enhancing. From the perspective of conservation of resources theory, strain-related job stress represents a condition in which valued resources are threatened, depleted, or insufficiently replenished [18, 20, 21]. When employees experience strain-related job stress, they are likely to allocate their remaining time, energy, and attention toward coping with immediate work demands rather than investing in discretionary and resource-intensive behaviors.

Innovative behavior is one such resource-intensive behavior. It involves not only generating novel ideas, but also promoting those ideas to others and implementing them in work settings [25, 41]. These activities require cognitive flexibility, attention, persistence, experimentation, and social engagement. Employees must recognize problems, process information, search for alternatives, persuade others, and overcome resistance during implementation. Strain-related job stress may interfere with these processes by consuming cognitive and emotional resources, narrowing attention, and reducing employees’ willingness to engage in exploration and experimentation [29, 37].

At the same time, it is important not to assume that all job demands are uniformly harmful. The challenge–hindrance stressor literature suggests that some demands may motivate employees by signaling opportunities for learning, achievement, or growth, whereas others constrain goal attainment and deplete resources [6, 28]. Accordingly, our argument is not that all work demands necessarily reduce innovative behavior. Rather, because the present study focuses on strain-related job stress involving psychological and physical tension, this form of stress is more closely aligned with resource depletion than motivational activation. Employees experiencing such stress should have fewer resources available for the sustained cognitive and social efforts required for innovative behavior. Accordingly, we expect strain-related job stress to be negatively related to employees’ innovative behavior.

- Hypothesis 1: Strain-related job stress is negatively related to employees’ innovative behavior.

### The mediating role of knowledge hiding behavior

Knowledge hiding is defined as the intentional attempt to withhold or conceal knowledge requested by another person [9]. Although knowledge hiding has often been discussed as a counterproductive or interpersonal behavior, COR theory suggests that it may also function as a defensive response to resource threat. Knowledge is a valuable work resource because it helps employees solve problems, maintain competence, exercise influence, and preserve status within the organization. However, sharing knowledge is not costless. Responding to others’ knowledge requests may require time, attention, cognitive effort, emotional patience, and the disclosure of expertise that employees may view as personally valuable [9, 10].

Under conditions of strain-related job stress, employees may become more protective of their remaining resources. When work demands are already experienced as taxing, requests for knowledge may be interpreted not merely as opportunities for cooperation but as additional resource claims. In such situations, employees may attempt to conserve resources by limiting the time, effort, and expertise they provide to others. Prior research has similarly suggested that resource threats and adverse workplace experiences can encourage knowledge hiding as a self-protective response [7, 17, 38, 44]. Thus, strain-related job stress may increase employees’ tendency to hide knowledge because knowledge hiding allows them to avoid further resource expenditure and retain control over valued knowledge.

Knowledge hiding, in turn, may be associated with lower levels of employees’ own innovative behavior. Innovative behavior depends heavily on knowledge exchange, feedback, reciprocal assistance, and collaborative problem solving [3, 25, 41]. Employees who hide knowledge may weaken these social processes in several ways. First, knowledge hiding may reduce reciprocity. Coworkers who perceive that an employee withholds knowledge may become less willing to provide information, feedback, or assistance in return. Second, knowledge hiding may limit idea recombination. Because innovative behavior often emerges from combining diverse perspectives and information, restricting knowledge exchange can reduce opportunities to refine and develop new ideas. Third, knowledge hiding may reinforce a defensive orientation that is inconsistent with openness, experimentation, and learning, all of which are important for innovative behavior.

Therefore, knowledge hiding may become a social-behavioral pathway through which strain-related job stress is associated with lower innovative behavior. Employees experiencing strain-related job stress may hide knowledge to conserve resources, but this defensive behavior may unintentionally weaken the reciprocal and collaborative knowledge flows needed for their own innovative behavior. Importantly, we do not argue that knowledge hiding is the only or dominant mechanism linking job stress to innovative behavior. Rather, we propose that knowledge hiding represents a theoretically meaningful pathway because it captures how strain-related job stress may alter employees’ knowledge-related social behavior, not merely their internal psychological state. Accordingly, we propose the following:

- Hypothesis 2: Knowledge hiding mediates the negative relationship between strain-related job stress and employees’ innovative behavior, such that strain-related job stress is positively related to knowledge hiding, which in turn is negatively related to innovative behavior.

### The moderating role of leader–member exchange quality

Leader–member exchange quality refers to the quality of the exchange relationship between supervisors and subordinates. High-quality leader–member exchange relationships are characterized by mutual trust, respect, obligation, understanding, and work-related assistance, whereas low-quality exchange relationships tend to be more transactional and limited in relational resources [16, 43]. This construct is broader than leader support narrowly defined. However, high-quality leader–member exchange can provide employees with supportive relational resources that are directly relevant to coping with stressful work demands, including supervisor understanding, guidance, influence, assistance, and interpersonal security.

From a COR perspective, leader–member exchange quality represents a relational resource that may reduce employees’ need to engage in defensive resource-protection behaviors. In high-quality exchange relationships, supervisors may provide emotional reassurance, clarify ambiguous demands, assist with workload management, and offer task-related guidance. These resources may help employees appraise stressful work demands as more manageable and less threatening to their remaining resources. In addition, high-quality leader–member exchange may signal that knowledge exchange is valued and safe, thereby reducing employees’ perceived need to protect themselves by withholding knowledge.

Leader–member exchange quality is particularly relevant to the relationship between strain-related job stress and knowledge hiding because knowledge requests often occur in ongoing social and task-related contexts. When leader–member exchange quality is high, employees experiencing strain-related job stress may feel that they have sufficient relational resources, guidance, and supervisory assistance to respond to coworkers’ knowledge requests without fearing further depletion. Under such conditions, employees may be less likely to interpret knowledge sharing as an excessive burden or threat. In contrast, when leader–member exchange quality is low, employees may experience strain-related job stress as more isolating and resource-depleting. Without sufficient supervisor understanding, assistance, or relational security, employees may be more likely to conserve their remaining resources by withholding knowledge.

Thus, leader–member exchange quality should weaken the positive association between strain-related job stress and knowledge hiding. This argument positions leader–member exchange quality not simply as a generally favorable supervisor–subordinate relationship, but as a relational resource that may interrupt the transition from resource threat to defensive knowledge regulation. Accordingly, we propose the following:

- Hypothesis 3: Leader–member exchange quality moderates the positive relationship between strain-related job stress and knowledge hiding, such that the relationship is stronger when leader–member exchange quality is low rather than high.

The preceding hypotheses suggest a conditional indirect process. Specifically, we propose that strain-related job stress is positively related to knowledge hiding because stressed employees may attempt to conserve threatened or depleted resources. We further propose that knowledge hiding is negatively related to innovative behavior because it weakens the reciprocal, collaborative, and knowledge-based processes that support idea generation, promotion, and implementation. However, this indirect relationship should depend on the level of leader–member exchange quality.

When leader–member exchange quality is low, employees facing strain-related job stress are likely to have fewer relational resources available to help them cope with work demands. In such situations, knowledge requests from others may be more readily perceived as additional burdens or threats to remaining resources. Employees may therefore be more likely to hide knowledge, and this knowledge hiding may subsequently limit the knowledge exchange and collaborative support needed for innovative behavior. As a result, the indirect relationship between strain-related job stress and innovative behavior through knowledge hiding should be stronger when leader–member exchange quality is low.

In contrast, when leader–member exchange quality is high, employees experiencing strain-related job stress may receive supervisor understanding, guidance, and work-related assistance. These relational resources may reduce the need to rely on knowledge hiding as a defensive coping strategy. Because high-quality leader–member exchange can help employees manage stress and maintain more open knowledge-related interactions, the indirect relationship between strain-related job stress and innovative behavior through knowledge hiding should be weaker under high leader–member exchange quality. Accordingly, we propose the following:

- Hypothesis 4: Leader–member exchange quality moderates the indirect relationship between strain-related job stress and innovative behavior through knowledge hiding, such that the indirect relationship is stronger when leader–member exchange quality is low rather than high.

## Method

### Participants and procedures

We collected two-wave time-lagged survey data from full-time employees in South Korea, with a two-week interval between the two survey waves. Participants were recruited through a reputable online survey firm that maintains a broad panel of South Korean working adults. To be eligible for participation, respondents were required to be currently employed full-time. Participants who completed both survey waves received monetary compensation equivalent to approximately US$5.

At Time 1, 839 participants completed measures of strain-related job stress, leader–member exchange quality, and demographic characteristics, including age, gender, education, and organizational tenure. Two weeks later, participants who completed the Time 1 survey were invited to complete the Time 2 survey, which assessed knowledge hiding and innovative behavior. A total of 541 participants completed both waves, yielding a retention rate of 64.48%. We excluded 45 participants who failed an attention check item. The final analytic sample consisted of 496 employees.

Among the final sample, 261 participants were male and 235 were female. The average age was 38.45 years (SD = 11.97). Regarding education, 30 participants had completed below high school, 100 had completed a two- or three-year college degree, 311 had completed a four-year university degree, 37 had a master’s degree, and 18 had a doctoral degree. In terms of organizational tenure, 135 participants had worked in their current organization for less than six months, 99 for more than six months to three years, 200 for more than three years to six years, 40 for more than six years to ten years, and 22 for more than ten years. Additional demographic information is presented in Table 1.

**Table 1:** Demographic characteristics of respondents

Demographic Characteristics		Frequency	%
Gender	Male	261	52.6%
	Female	235	47.4%
Age (years) (M = 38.45, SD = 11.97)	10–19 years old	3	0.6%
	20–29 years old	125	25.2%
	30–39 years old	166	33.5%
	40–49 years old	111	22.4%
	More than 50 years old	91	18.3%
Education	Below high school	30	6%
	2–3 years college	100	20.2%
	4 years university	311	62.7%
	Master’s degree	37	7.5%
	Doctoral degree	18	3.6%
Organizational tenure	Less than 6 months	135	27.2%
	More than 6 months to 3 years	99	20%
	More than 3 years to 6 years	200	40.3%
	More than 6 years to 10 years	40	8.1%
	More than 10 years	22	4.4%

### Measures

All focal constructs were measured using established scales. Unless otherwise noted, participants responded to all items using a seven-point Likert-type scale ranging from 1 = strongly disagree to 7 = strongly agree.

#### Strain-related job stress

 Strain-related job stress was measured at Time 1 using 11 items from Parker and DeCotiis’s [31] job stress scale. The original scale includes 13 items; however, two items were excluded because they showed very low factor loadings in the measurement model (0.11 and 0.05). The retained 11 items capture employees’ psychological and physical tension associated with work, rather than challenge demands or motivating job demands. Sample items include “My job gets to me more than it should” and “Sometimes when I think about my job, I get a tight feeling in my chest.”

#### Knowledge hiding

Knowledge hiding was measured at Time 2 using items from Connelly et al., [9]. Knowledge hiding refers to employees’ intentional withholding or concealment of knowledge requested by others. Sample items include “I pretended that I did not know the information during work” and “I pretended to help coworkers but instead gave them information different from what they wanted.”

#### Innovative behavior

Innovative behavior was measured at Time 2 using a six-item scale from Scott and Bruce [41]. The scale captures employees’ generation, promotion, and implementation of new ideas. Sample items include “I generate creative ideas” and “I develop adequate plans and schedules for the implementation of new ideas.”

#### Leader–member exchange quality

Leader–member exchange quality was measured at Time 1 using the seven-item leader–member exchange scale originally reported by Scandura and Graen [39] and later established in the LMX literature by Graen and Uhl-Bien [16]. The scale assesses employees’ perceived quality of the exchange relationship with their immediate supervisor. Consistent with the broader LMX construct, the items capture aspects of supervisor understanding, work-related assistance, influence, and the quality of the supervisor–subordinate relationship. Sample items include “My supervisor understands my problems and needs” and “My supervisor uses his/her power to help me solve problems in my work.”

#### Control variables

We controlled for age, gender, education, and organizational tenure because these demographic characteristics may be associated with employees’ innovative behavior and workplace knowledge-related behavior. Age and organizational tenure were measured in years or tenure categories, and gender and education were coded according to the response categories reported in Table 1.

## Results

Before testing the hypotheses, we conducted confirmatory factor analysis to examine the distinctiveness of the focal constructs: strain-related job stress, leader–member exchange quality, knowledge hiding, and innovative behavior. The hypothesized four-factor model showed acceptable fit to the data, χ²(241) = 918.29, CFI = 0.94, TLI = 0.93, SRMR = 0.04, RMSEA = 0.03. The four-factor model also fit the data significantly better than the alternative three-factor model, Δχ² = 237.44, *p* < .001, supporting the discriminant validity of the focal constructs.

As shown in Table 2, the composite reliability and Cronbach’s alpha values for the focal constructs exceeded the recommended threshold of 0.70, indicating acceptable internal consistency. The average variance extracted values also exceeded the recommended 0.50 threshold, providing evidence of convergent validity [13]. Means, standard deviations, reliabilities, and correlations are presented in Table 3.

**Table 2:** Convergent validity results

Construct	Factor Loadings	AVE	CR
Strain-Related Job Stress	0.82, 0.78, 0.85, 0.88, 0.89, 0.84, 0.93, 0.91, 0.90, 0.92, 0.92	0.82	0.92
Leader–Member Exchange Quality	0.93, 0.91, 0.90, 0.89, 0.88, 0.87, 0.92	0.87	0.94
Knowledge Hiding	0.85, 0.88, 0.91, 0.82, 0.86, 0.92, 0.81, 0.84, 0.85, 0.88, 0.89, 0.90, 0.87, 0.89, 0.85, 0.91	0.88	0.95
Innovative Behavior	0.90, 0.88, 0.89, 0.91, 0.87, 0.92	0.89	0.93

**Table 3:** Descriptive statistics and correlations

	Mean	SD	1	2	3	4	5	6	7	8
1. Gender	0.52	0.49								
2. Age	38.45	11.97	0.02							
3. Education	2.82	0.79	− 0.04	− 0.06						
4. Tenure	2.42	1.10	− 0.14^**^	− 0.10^*^	0.08					
5. Strain-Related Job Stress	2.67	0.78	− 0.04	− 0.08^*^	0.17^***^	0.24^***^	(0.86)			
6. LMX Quality	4.86	1.31	0.04	0.01	− 0.13^**^	− 0.09^*^	− 0.28^***^	(0.93)		
7. Knowledge Hiding	2.58	1.04	− 0.07	− 0.14^**^	0.06	0.21^***^	0.18^***^	− 0.35^***^	(0.96)	
8. Innovative Behavior	3.42	1.00	− 0.01	0.08	− 0.11^*^	− 0.01	− 0.32^***^	0.31^***^	− 0.26^***^	(0.90)

*N* = 496, Reliability is placed along the diagonal in parentheses

^*^p < .05, ^**^p < .01, ^***^p < .001

Because all focal variables were collected using self-report survey measures, common method bias was a potential concern. We attempted to reduce this concern procedurally by separating the measurement of strain-related job stress and leader–member exchange quality from the measurement of knowledge hiding and innovative behavior by two weeks, assuring participants of confidentiality, and including an attention check. We also conducted Harman’s single-factor test. The first unrotated factor accounted for 29.96% of the variance, below the commonly used 50% criterion. However, because Harman’s single-factor test has important limitations, these results should not be interpreted as fully eliminating the possibility of common method bias. We therefore return to this issue in the limitations section.

Hypothesis 1 predicted that strain-related job stress would be negatively related to innovative behavior. As shown in Model 4 of Table 4, strain-related job stress was negatively and significantly associated with innovative behavior, b = − 0.34, SE = 0.05, *p* < .001. Thus, Hypothesis 1 was supported.

**Table 4:** Results of hierarchical regression analyses

Variables	Knowledge Hiding				Innovative Behavior	
	Model 1	Model 2	Model 3		Model 4	Model 5
Constant	2.16 (0.31)^***^	3.82 (0.37)^***^	2.83 (0.60)^***^		3.27 (0.36)^***^	3.88 (0.39)^***^
Age	–0.01 (0.00)^**^	–0.01 (0.00)^**^	–0.01 (0.00)^**^		0.00 (0.00)	0.00 (0.00)
Gender	–0.07 (0.09)	–0.05 (0.08)	–0.08 (0.08)		–0.05 (0.06)	–0.06 (0.08)
Education	0.02 (0.05)	–0.00 (0.05)	–0.02 (0.05)		–0.04 (0.05)	–0.04 (0.05)
Organizational tenure	0.15 (0.04)^***^	0.15 (0.04)^***^	0.14 (0.04)^**^		0.07 (0.03)	0.09 (0.03)^*^
Strain-related job stress	0.17 (0.06)^**^	0.06 (0.05)^**^	0.44 (0.19)^*^		–0.34 (0.05)^***^	–0.33 (0.05)^***^
LMX quality		–0.25 (0.03)^***^	–0.05 (0.10)		0.18 (0.03)^***^	0.14 (0.03)^***^
Strain-related job stress × LMX quality			–0.07 (0.03)^*^			
Knowledge hiding						–0.15 (0.04)^***^
*R* ^2^	0.08^***^	0.17^***^	0.18^**^		0.17^***^	0.19^***^
∆*R*^2^		0.09^***^	0.01^*^			0.02^**^

*N* = 496. Unstandardized regression coefficients are reported, with standard errors in parentheses

^*^p < .05, ^**^p < .01, ^***^p < .001

Hypothesis 2 predicted that knowledge hiding would mediate the negative relationship between strain-related job stress and innovative behavior. We tested this indirect relationship using a bootstrap approach [36]. As shown in Model 1 of Table 4, strain-related job stress was positively associated with knowledge hiding, b = 0.17, SE = 0.06, *p* < .01. As shown in Model 5, knowledge hiding was negatively associated with innovative behavior, b = − 0.15, SE = 0.04, *p* < .001, controlling for strain-related job stress and the control variables. The bootstrapped indirect relationship between strain-related job stress and innovative behavior through knowledge hiding was significant, indirect effect = − 0.03, SE = 0.01, 95% CI [-0.07, − 0.01]. Because the direct relationship between strain-related job stress and innovative behavior remained significant after including knowledge hiding, b = − 0.33, SE = 0.05, *p* < .001, the results suggest partial mediation. Thus, Hypothesis 2 was supported.

Hypothesis 3 predicted that leader–member exchange quality would moderate the positive relationship between strain-related job stress and knowledge hiding, such that the relationship would be stronger when leader–member exchange quality was low rather than high. As shown in Model 3 of Table 4, the interaction between strain-related job stress and leader–member exchange quality was negatively and significantly associated with knowledge hiding, b = − 0.07, SE = 0.03, *p* < .05. To interpret this interaction, we conducted simple slopes analyses following Aiken and West [1]. As shown in Table 5, strain-related job stress was positively related to knowledge hiding when leader–member exchange quality was low, b = 0.18, SE = 0.08, 95% CI [0.02, 0.35]. However, this relationship was not significant when leader–member exchange quality was at the mean level, b = 0.08, SE = 0.06, 95% CI [-0.02, 0.20], or when leader–member exchange quality was high, b = − 0.00, SE = 0.06, 95% CI [-0.14, 0.12].

**Table 5:** Conditional effects of strain-related job stress on knowledge hiding at values of leader–member exchange quality

Leader–Member Exchange Quality	Conditional effects		
	Effect	SE	95% CI (LLCI, ULCI)
Low (mean – 1*SD*)	0.18	0.08	[0.02, 0.35]
Mean	0.08	0.06	[-0.02, 0.20]
High (mean + 1*SD*)	− 0.00	0.06	[-0.14, 0.12]

*N* = 496. Values represent the conditional effects of strain-related job stress on knowledge hiding at low, mean, and high levels of LMX quality

*LLCI* Lower limit confidence interval, *ULCI* Upper limit confidence interval

**Fig. 2:**
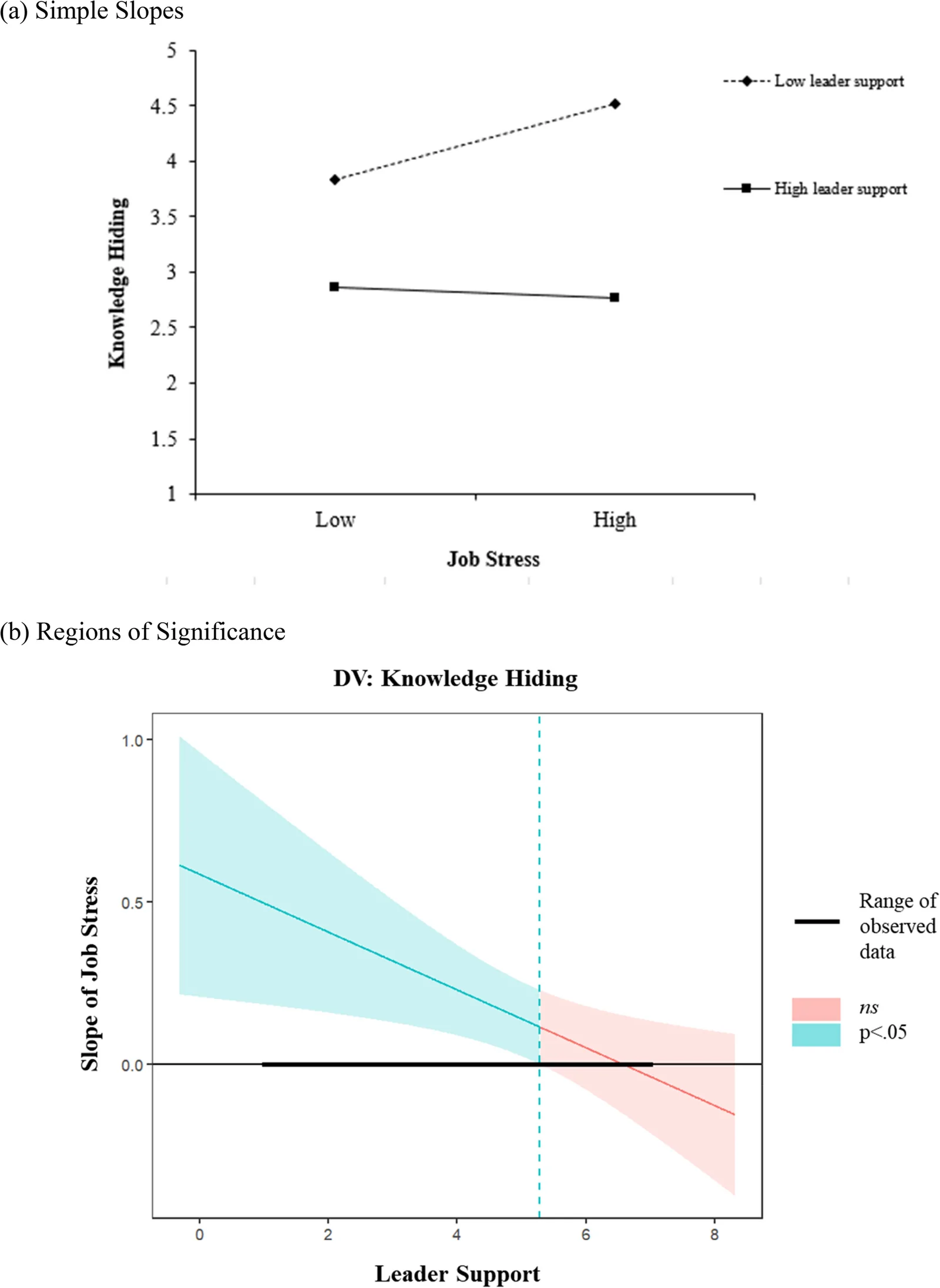
Interaction of job stress and leader support on knowledge hiding. Note. Graph **a **= simple slope analysis of the moderating effect. Graph **b **= regions of significance analysis of the moderating effect (the range of observed values of leader support is [1.00, 7.00])

Figure 2 graphically illustrates this interaction pattern. As shown in Fig. 2a, the positive relationship between strain-related job stress and knowledge hiding was evident when leader–member exchange quality was low. In contrast, the slope became substantially weaker and nearly flat when leader–member exchange quality was high. The regions-of-significance analysis shown in Fig. 2b further indicated that the conditional effect of strain-related job stress on knowledge hiding was statistically significant only at relatively low levels of leader–member exchange quality within the observed range. Taken together, these findings indicate that leader–member exchange quality weakened the positive association between strain-related job stress and knowledge hiding. Thus, Hypothesis 3 was supported.

Hypothesis 4 predicted that leader–member exchange quality would moderate the indirect relationship between strain-related job stress and innovative behavior through knowledge hiding, such that the indirect relationship would be stronger when leader–member exchange quality was low rather than high. We tested this conditional indirect relationship using a bootstrapping approach with 10,000 resamples. The results supported this conditional indirect pattern. Specifically, the indirect relationship between strain-related job stress and innovative behavior through knowledge hiding was significant when leader–member exchange quality was low, indirect effect = − 0.03, SE = 0.01, 95% CI [-0.05, − 0.00], but was not significant when leader–member exchange quality was at the mean level, indirect effect = − 0.01, SE = 0.01, 95% CI [-0.03, 0.01], or high, indirect effect = 0.00, SE = 0.01, 95% CI [-0.02, 0.02]. In addition, the estimated index of moderated mediation was positive, index = 0.01, SE = 0.01, 95% CI [0.00, 0.02], indicating that the indirect relationship between strain-related job stress and innovative behavior through knowledge hiding was stronger under low leader–member exchange quality than under high leader–member exchange quality. Thus, Hypothesis 4 was supported.

### Discussion and implications

### Discussion

Drawing on conservation of resources theory, this study examined how strain-related job stress is associated with employees’ innovative behavior through knowledge hiding and whether leader–member exchange quality weakens this process. Using two-wave time-lagged data from full-time employees in South Korea, we found that strain-related job stress was negatively associated with innovative behavior and positively associated with knowledge hiding. Knowledge hiding, in turn, was negatively associated with innovative behavior and partially mediated the relationship between strain-related job stress and innovative behavior. In addition, leader–member exchange quality weakened the positive relationship between strain-related job stress and knowledge hiding, such that this relationship was significant when leader–member exchange quality was low but not when it was at the mean or high level.

These findings suggest that strain-related job stress may be associated with lower innovative behavior not only through employees’ internal psychological and cognitive resource depletion, but also through changes in how employees regulate access to their knowledge. Under stressful work conditions, employees may become more protective of their time, effort, attention, and expertise. As a result, they may respond to knowledge requests defensively by withholding or concealing knowledge. Although such behavior may help employees conserve resources in the short term, it may also weaken the reciprocal and collaborative knowledge flows needed for their own innovative behavior. Given the modest size of the indirect effect, however, knowledge hiding should be interpreted as one partial social-behavioral pathway rather than as the primary explanation for the relationship between strain-related job stress and innovative behavior.

The findings also highlight the importance of leader–member exchange quality in this defensive process. Leader–member exchange quality appears to matter not simply because high-quality supervisor–subordinate relationships are generally beneficial, but because they provide relational resources that may make defensive knowledge regulation less necessary. Although leader–member exchange quality is broader than leader support narrowly defined, high-quality exchange relationships can provide employees with supervisor understanding, work-related guidance, assistance, and interpersonal security. When employees experience high-quality leader–member exchange, strain-related job stress is less strongly associated with knowledge hiding. In contrast, when leader–member exchange quality is low, employees may experience strain-related job stress as more isolating and threatening, making knowledge hiding a more likely resource-protection response.

Overall, the present study advances a more socially embedded understanding of the stress–innovative behavior relationship. Rather than viewing strain-related job stress only as an internal strain process that reduces motivation or cognitive capacity, our findings suggest that stress may also shape employees’ knowledge-related social behavior. This perspective is important because innovative behavior depends not only on what employees know, but also on whether they remain willing and able to participate in reciprocal knowledge exchange under stressful conditions. At the same time, because the study used a two-wave observational design and self-report measures, the findings should be interpreted as evidence of time-lagged associations rather than definitive causal effects.

### Theoretical implications

This study offers several theoretical implications for research on strain-related job stress, knowledge hiding, innovative behavior, and leader–member exchange quality. First, the study contributes to the job stress and innovative behavior literature by identifying knowledge hiding as a social-behavioral pathway linking strain-related job stress to lower innovative behavior. Prior research has often explained the negative relationship between job stress and innovative behavior through internal mechanisms such as resource depletion, emotional exhaustion, cognitive overload, and reduced motivation [4, 20, 29, 37]. These explanations are important, but they primarily focus on what stress does to employees’ internal psychological and cognitive states. Our findings extend this literature by suggesting that strain-related job stress may also alter employees’ knowledge-related social behavior. This extension is important because innovative behavior depends not only on individual creativity or motivation, but also on access to information, feedback, collaboration, and knowledge recombination [2, 3, 25, 41]. Thus, the present study suggests that strain-related job stress may be associated with lower innovative behavior partly by encouraging employees to regulate knowledge access more defensively.

Second, this study contributes to the knowledge hiding literature by conceptualizing knowledge hiding as a potential resource-protection response under strain-related job stress. Prior research has typically treated knowledge hiding as a counterproductive interpersonal behavior that damages trust, creativity, collaboration, and knowledge exchange [7, 8, 9, 42]. Our study does not challenge this view, but it adds a complementary perspective. Drawing on conservation of resources theory, we suggest that knowledge hiding may also emerge when employees attempt to conserve valued resources such as time, attention, energy, and expertise under stressful conditions [17, 18, 20, 44]. This perspective helps explain why employees may hide knowledge even when doing so can ultimately be associated with lower levels of their own innovative behavior. Knowledge hiding may provide short-term resource protection, but it may also reduce the reciprocal and collaborative knowledge flows needed for innovative behavior.

Third, the study extends conservation of resources theory by applying it more specifically to knowledge-related behavior. COR theory has been widely used to explain why stress produces strain and reduces positive work outcomes [18, 19, 20, 21]. However, broad applications of COR can become overly general unless they clarify which resources are threatened, which resources are protected, and how employees attempt to conserve them. In the present study, strain-related job stress represents a resource threat, knowledge represents a valued work resource, knowledge hiding represents a defensive conservation response, innovative behavior represents a resource-intensive outcome, and leader–member exchange quality represents a relational resource that may reduce the need for defensive protection. This construct-specific application responds to calls for more precise theorizing about resource dynamics in organizational behavior [18]. It also shows that COR theory can explain not only internal strain responses, but also employees’ social decisions about whether to share or withhold knowledge.

Fourth, this study contributes to leadership and stress research by clarifying why leader–member exchange quality is relevant to the relationship between strain-related job stress and knowledge hiding. Unlike leader support narrowly defined, leader–member exchange quality captures the broader quality of the supervisor–subordinate exchange relationship, including mutual trust, respect, obligation, understanding, and work-related assistance [16, 43]. From a COR perspective, high-quality leader–member exchange can function as a relational resource that provides employees with supervisor guidance, assistance, and interpersonal security when they face stressful work demands. Our findings suggest that leader–member exchange quality weakens the positive relationship between strain-related job stress and knowledge hiding. This indicates that high-quality leader–member exchange may matter not simply because it reflects a generally favorable supervisor–subordinate relationship, but because it may interrupt the transition from resource threat to defensive knowledge regulation.

Finally, this study contributes to research on innovative behavior by suggesting that knowledge hiding may be associated with lower innovative behavior for the focal employee, not only for coworkers or teams. Prior research has emphasized that knowledge hiding can damage others’ creativity and broader organizational knowledge exchange [7, 8, 42]. Our findings suggest that employees who hide knowledge may also limit their own innovative behavior by weakening reciprocal exchange, reducing access to feedback, and restricting opportunities for idea recombination. This logic is consistent with social exchange theory and reciprocity-based views of workplace relationships, which suggest that employees’ willingness to provide resources to others can shape the resources they receive in return [5, 11, 15]. Given the modest size of the indirect effect, however, this pathway should be understood as one partial mechanism rather than as a comprehensive explanation for the relationship between strain-related job stress and innovative behavior.

### Practical implications

The findings also offer several practical implications for organizations, managers, and human resource practitioners. First, organizations seeking to promote employees’ innovative behavior should recognize strain-related job stress as more than an individual well-being issue. Strain-related job stress may also shape how employees participate in knowledge exchange. Prior research has shown that stress and job demands can reduce employees’ energy, engagement, creativity, and innovative behavior when they exceed available resources [4, 29, 37, 40]. Our findings suggest that employees experiencing strain-related job stress may also become more defensive about sharing knowledge. Therefore, stress management practices may support innovative behavior not only by reducing strain, but also by preserving employees’ willingness to participate in open knowledge exchange. Organizations can reduce unnecessary stress by clarifying roles, monitoring workload, improving staffing adequacy, providing realistic deadlines, and ensuring that employees have sufficient resources to meet job demands.

Second, organizations should treat knowledge hiding as a potential hidden barrier to innovative behavior. Because knowledge hiding is intentional and often difficult to observe, managers may not immediately recognize it as a problem [9, 10]. Employees may appear cooperative while delaying responses, providing incomplete information, or pretending not to know. Such behaviors may undermine trust, reduce collaboration, and weaken the knowledge flows needed for creativity and innovation [7, 8, 42]. To reduce knowledge hiding, organizations should build norms of reciprocity, trust, and psychological safety. Practices such as mentoring programs, peer learning sessions, cross-functional meetings, and knowledge-sharing platforms may help, but they are likely to be most effective when employees believe that sharing knowledge will not expose them to exploitation, excessive workload, or loss of control.

Third, the findings suggest that high-quality leader–member exchange relationships may be especially important when employees are under stress. Supervisors should not assume that employees experiencing strain-related job stress simply need to work harder or manage their time better. High-quality leader–member exchange relationships may reduce defensive knowledge hiding by providing employees with supervisor understanding, guidance, assistance, and interpersonal security. These relational resources may help employees prioritize tasks, clarify expectations, manage workload demands, and feel safer engaging in knowledge exchange. Leader development programs should therefore emphasize not only general empathy and communication, but also relationship-building behaviors that strengthen trust, mutual respect, timely feedback, role clarification, and work-related assistance.

Fourth, organizations should train leaders to identify early signs of defensive knowledge regulation. Employees under stress may not openly state that they are hiding knowledge. Instead, they may delay responses, avoid collaboration, provide minimal information, or become less available to coworkers. Because knowledge hiding can be subtle and difficult to detect, leaders should avoid immediately interpreting these behaviors as poor attitude or lack of commitment. Instead, they should examine whether employees are experiencing excessive demands, unclear expectations, weak supervisor–subordinate relationships, or fear that sharing knowledge will create additional burdens. Addressing the underlying resource threat and relational context may be more effective than simply urging employees to share more knowledge.

Finally, the findings suggest that innovation initiatives should integrate stress management, knowledge-sharing systems, and leader–member relationship development. Many organizations attempt to encourage innovation by promoting creativity, idea generation, or knowledge-sharing platforms. However, such initiatives may be less effective if employees are working under stressful conditions that make them defensive about their time, energy, and expertise. Research on creativity and innovation has consistently emphasized the importance of supportive contexts, sufficient resources, and open information exchange [2, 3, 41]. Therefore, organizations should create work environments in which employees have enough resources to engage in both their own tasks and collaborative knowledge exchange. By reducing unnecessary stress, discouraging knowledge hiding, and strengthening high-quality leader–member exchange relationships, organizations may be better able to sustain employees’ innovative behavior even under demanding work conditions.

### Limitations and future research

Although this study contributes to research on strain-related job stress, knowledge hiding, innovative behavior, and leader–member exchange quality, several limitations should be acknowledged. First, although we used a two-wave time-lagged design, the present study does not allow strong causal inference. Strain-related job stress and leader–member exchange quality were measured at Time 1, whereas knowledge hiding and innovative behavior were measured two weeks later. This temporal separation is an improvement over a purely cross-sectional design and helps reduce some concerns about simultaneous measurement. However, the two-week interval may have captured relatively short-term associations rather than the longer developmental process through which stress accumulates, knowledge hiding becomes patterned, and innovative behavior changes over time. These relationships may also be reciprocal. For example, employees who engage in knowledge hiding may later experience lower collaboration, reduced support, or greater stress, and employees with lower innovative behavior may perceive their work environment more negatively. Therefore, the findings should be interpreted as time-lagged associations rather than definitive evidence of causal effects. Future research should use multi-wave longitudinal designs with longer time intervals, such as two to three months, cross-lagged panel models, or experience sampling methods to examine how strain-related job stress, knowledge hiding, leader–member exchange quality, and innovative behavior influence one another over time [14, 33].

Second, common method bias remains a potential concern because all focal variables were measured using self-report surveys. Although we used temporal separation, ensured respondent anonymity, included an attention check, and conducted confirmatory factor analyses and Harman’s single-factor test, these procedures cannot fully rule out common method variance [34, 35]. This issue is particularly relevant because knowledge hiding is a socially undesirable behavior and innovative behavior may be vulnerable to self-enhancement bias. Employees may underreport their own knowledge hiding or overreport their innovative behavior. Future research should therefore incorporate multi-source data, such as supervisor-rated innovative behavior, coworker-rated knowledge hiding, or objective indicators of knowledge contribution and innovation-related outcomes. Experimental or mixed-method designs could also help clarify whether strain-related job stress is associated with defensive knowledge regulation and whether leader–member exchange quality weakens this association.

Third, although the study focused on knowledge hiding as a theoretically meaningful social-behavioral pathway, knowledge hiding is unlikely to be the only mechanism linking strain-related job stress to innovative behavior. Prior research has suggested that stress may impair innovative behavior through emotional exhaustion, burnout, cognitive overload, reduced intrinsic motivation, lower work engagement, and diminished psychological safety [4, 29, 37]. Our study identifies knowledge hiding as one pathway through which strain-related job stress is associated with lower innovative behavior, but future research should compare knowledge hiding with these alternative mediating mechanisms. Such work would help clarify whether knowledge hiding explains unique variance beyond internal strain-based mechanisms and whether multiple pathways operate simultaneously or sequentially.

Fourth, the measurement of knowledge hiding deserves further attention. Knowledge hiding has often been conceptualized as a multidimensional construct consisting of evasive hiding, playing dumb, and rationalized hiding [9]. The present study examined knowledge hiding as an overall tendency to withhold or conceal requested knowledge. This approach is consistent with our theoretical focus on defensive knowledge regulation under stress, but it may obscure potentially important differences among knowledge hiding forms. For example, strain-related job stress may be more strongly related to evasive hiding or playing dumb than to rationalized hiding, because some forms of hiding may be more defensive whereas others may be more normatively justified. Future research could examine whether different dimensions of knowledge hiding have distinct antecedents, motives, and consequences for innovative behavior.

Fifth, the composition of the sample should be considered when interpreting the findings. In particular, 27.2% of the final analytic sample had worked in their current organization for less than six months. Employees with very short tenure may differ from longer-tenured employees in their knowledge base, social integration, and familiarity with organizational routines. For such employees, lower knowledge provision may sometimes reflect limited knowledge, uncertainty, or ongoing learning rather than intentional concealment. Although our measure captured self-reported knowledge hiding, this sample characteristic suggests that future research should examine whether organizational tenure moderates the relationship between strain-related job stress and knowledge hiding. Future studies could also distinguish more clearly between employees’ lack of knowledge, inability to share knowledge, and intentional decisions to hide knowledge.

Sixth, the sample consisted of full-time employees in South Korea who were recruited through an online survey panel. Although this context is useful for examining supervisor–subordinate dynamics in a relatively hierarchical and relationally embedded work culture, it may also limit the generalizability of the findings. Korean employees’ responses to strain-related job stress, knowledge requests, and leader–member exchange quality may be shaped by cultural values related to hierarchy, collectivism, relational obligations, and supervisor authority [12, 22]. Therefore, the relationships observed in this study may differ in cultural contexts with lower power distance, more individualistic work norms, or different expectations regarding supervisor–subordinate relationships. Future research should test the model in other national and cultural settings and examine whether cultural values moderate the relationships among strain-related job stress, knowledge hiding, leader–member exchange quality, and innovative behavior.

Seventh, although participants were recruited from diverse industries, the present study did not explicitly model industry, occupation, or organizational nesting. Employees working in different industries may experience different levels of strain-related job stress, knowledge interdependence, innovation requirements, and supervisor–subordinate relationship quality. For example, knowledge hiding may have stronger implications for innovative behavior in knowledge-intensive or highly interdependent jobs than in more routine work settings. Future research could use multilevel designs that account for employees nested within teams, organizations, or industries. Such research could also examine contextual moderators such as team climate, psychological safety, knowledge-sharing norms, and organizational support for innovation.

Finally, the observed indirect relationship between strain-related job stress and innovative behavior through knowledge hiding should be interpreted cautiously in terms of effect size. Although the indirect relationship was statistically significant, the magnitude of the effect was modest. This suggests that knowledge hiding is a meaningful but partial explanation of the stress–innovative behavior link. Future research should examine boundary conditions under which this pathway becomes stronger or weaker, such as task interdependence, job autonomy, competitive climate, psychological safety, or leader–member exchange quality. Examining these conditions would help determine when stress-related knowledge hiding is especially likely to be associated with lower employee innovative behavior.

## Conclusion

This study examined whether strain-related job stress is associated with lower employees’ innovative behavior through knowledge hiding and whether leader–member exchange quality weakens this process. Drawing on conservation of resources theory, we proposed that strain-related job stress may place employees in a resource-protection mode, making them more likely to defensively regulate access to their knowledge. Using two-wave time-lagged survey data from full-time employees in South Korea, we found that strain-related job stress was positively associated with knowledge hiding and negatively associated with innovative behavior. Knowledge hiding partially mediated the relationship between strain-related job stress and innovative behavior, and leader–member exchange quality weakened the positive relationship between strain-related job stress and knowledge hiding, such that this relationship was significant when leader–member exchange quality was low but not when it was at the mean or high level.

These findings suggest that strain-related job stress may be associated with lower innovative behavior not only through employees’ internal psychological resource depletion, but also through how employees manage knowledge exchange with others. Knowledge hiding appears to be a social-behavioral pathway through which stressed employees may protect their resources in the short term while unintentionally weakening the reciprocal and collaborative processes needed for innovative behavior. The findings also highlight high-quality leader–member exchange as a relational resource that may reduce employees’ need to engage in defensive knowledge regulation under stress.

Overall, this study contributes to a more socially embedded understanding of the stress–innovative behavior relationship. By integrating strain-related job stress, knowledge hiding, leader–member exchange quality, and innovative behavior within a COR framework, the study suggests that sustaining innovative behavior requires more than encouraging creativity or reducing stress in general. Organizations may also need to create relationally supportive conditions in which employees feel sufficiently resourced and secure to participate in open knowledge exchange, even under demanding work conditions.

## Data Availability

The datasets used and/or analyzed during the current study are available from the corresponding author upon reasonable request.
